# Preliminary Treatment by Exogenous 24-Epibrassinolide Influences Burning-Induced Electrical Signals and Following Photosynthetic Responses in Pea (*Pisum sativum* L.)

**DOI:** 10.3390/plants13233292

**Published:** 2024-11-23

**Authors:** Ekaterina Sukhova, Lyubov Yudina, Elizaveta Kozlova, Vladimir Sukhov

**Affiliations:** Department of Biophysics, N.I. Lobachevsky State University of Nizhny Novgorod, 603950 Nizhny Novgorod, Russia; n.catherine@inbox.ru (E.S.); lyubovsurova@mail.ru (L.Y.); elizagreen21@gmail.com (E.K.)

**Keywords:** 24-epibrassinolide, electrical signals, variation potential, photosynthesis, transpiration, pea

## Abstract

Long-distance electrical signals (ESs) are an important mechanism of induction of systemic adaptive changes in plants under local action of stressors. ES-induced changes in photosynthesis and transpiration play a key role in these responses increasing plant tolerance to action of adverse factors. As a result, investigating ways of regulating electrical signaling and ES-induced physiological responses is a perspective problem of plant electrophysiology. The current work was devoted to the analysis of the influence of preliminary treatment (spraying) by exogenous 24-epibrassinolide (EBL) on burning-induced ESs and following photosynthetic and transpiratory responses in pea (*Pisum sativum* L.). It was shown that preliminary treatment by 1 µM EBL (1 day before the experiment) increased the amplitude of burning-induced ESs (variation potentials) in leaves and decreased the time of propagation of these signals from the stem to the leaf. The EBL treatment weakly influenced the magnitudes of burning-induced decreasing the photosynthetic linear electron flow and CO_2_ assimilation, but these changes were accelerated. Burning-induced changes in the cyclic electron flow around photosystem I were also affected by the EBL treatment. The influence of the EBL treatment on burning-induced changes in the stomatal water conductance was not observed. Our results show that preliminary treatment by EBL can be used for the modification of electrical signals and following photosynthetic responses in plants.

## 1. Introduction

Under environmental conditions, terrestrial plants are often affected by spatially heterogeneous action of stressors. The generation and propagation of electrical signals (ESs) induced by the local action of these stressors is a mechanism for forming a plant systemic adaptive response including numerous fast physiological changes [1,2,3,4,5,6,7,8,9,10]. The activation of gene expression [11,12,13,14], stimulation of the production of stress phytohormones [14,15,16,17], increase in respiratory rate [18,19,20], suppression of phloem mass-flow [21,22,23], decrease in plant growth [24,25], and other changes can be induced by the ES propagation in terrestrial plants. 

Photosynthesis and transpiration are other important targets of ESs [2,3,6,8,10]. It is known that ESs suppress the photosynthetic CO_2_ assimilation (A_hv_) [26,27,28] and linear electron flow (LEF) [29], decrease the mesophyll conductance for CO_2_ [30], and activate the non-photochemical quenching of chlorophyll fluorescence [19,31,32,33,34] and cyclic electron flow around photosystem I (CEF) [29] in non-irritated leaves of plants. Changes in transpiration, which are also observed in non-irritated leaves after the ESs induction, are intricate because increasing [26,35,36] or decreasing [15,26,36] stomatal water conductance (G_H2O_) can be induced by localized plant irritation. Dynamics of changes in G_H2O_ can be similar to dynamics of changes in photosynthetic parameters [15,36] or differ from these dynamics [27,35]. Earlier, we hypothesized [37] that changes in G_H2O_ are strongly related to photosynthetic changes under limiting CO_2_ flux into the chloroplast stroma by the stomatal CO_2_ conductance, which is proportional to G_H2O_; in contrast, this relationship should be absent under limiting the CO_2_ flux by the mesophyll conductance for CO_2_. 

Both ES-induced photosynthetic and transpiration changes are probable to participate in increasing the plant tolerance to action of adverse factors [8,10]; particularly, electrical signals can stimulate plant tolerance (including tolerance of photosynthetic machinery) to the increased temperature, excess light, or soil drought [17,38,39,40,41,42,43]. 

Mechanisms of induction of changes in photosynthesis and transpiration are related to mechanisms of the ESs generation [8,10,37]. Three types of ESs are known: variation potential (VP) [25,44,45], action potential [1,2,46,47,48], and system potential [49,50]. 

VP is a long-term depolarization signal with an irregular shape, which is induced by local damages (e.g., burning, heating, or mechanical wounding) [1,3,10,45]. Transient inactivation of H^+^-ATPase in the plasma membrane, which is induced by the Ca^2+^ influx, is considered to be the main mechanism of VP generation [8,10]. The VP propagation is probable to be related to the transmission of the chemical agent (e.g., H_2_O_2_ or glutamate) [5,51,52] or hydraulic wave [44,53], which activates ligand-dependent or mechanical Ca^2+^ channels, respectively, in the plasma membrane. Interaction between chemical and hydraulic signals is also considered as a probable mechanism of VP propagation in some works [54,55,56]. 

The action potential is a self-propagating short-term depolarization spike [1,2,3,4,46], which is induced by non-damaging irritations (e.g., light [57,58], cooling [47], or touch [48,59]); its generation is in accordance with the “all-or-none” law. AP generation is mainly related to the transient activation of depolarization-dependent Ca^2+^, Cl^−^, and K^+^ channels in the plasma membrane [2,3,60]; however, the Ca^2+^-dependent short-term inactivation of H^+^-ATPase also participates in this generation [10].

Finally, system potential is a long-term hyperpolarization signal, which can be induced by the local action of various damaging and non-damaging stressors [49,50] including heating to moderate temperatures, illumination, or their combination [43,61]. Mechanisms of system potential are discussed; earlier, we hypothesized [10] that the signals are caused by the propagation of hydraulic signals with low magnitudes, moderate activation of Ca^2+^ channels in the plasma membrane, and the inactivation of inwardly rectifying K^+^ channels and H^+^-ATPase. 

Thus, the transient inactivation of H^+^-ATPase in the plasma membrane seems to be the common mechanism for the generation of variation potential, action potential, and system potential [10]; therefore, it can be hypothesized that this inactivation and changes in intra- and extracellular pH, which are caused by the inactivation, are the main reasons of ES-induced photosynthetic responses and increasing tolerance to action of adverse factors [8,10,37]. There are arguments supporting this hypothesis (see our review [10]): (i) dependence of magnitudes of ES-induced photosynthetic responses on the initial H^+^-ATPase activity; (ii) imitation of these responses under artificial inactivation of H^+^-ATPase in protoplasts, under induction of H^+^ influx in plant cells, and under acidification of the chloroplast medium (for isolated chloroplasts); and (iii) relationships between changes in intra- and extracellular pH and changes in photosynthetic parameters. The transient inactivation of H^+^-ATPase and activation of ion channels caused by this inactivation are also probable to be the mechanism of ES-induced changes in stomata opening [10]. 

Considering the positive influence of ESs and following photosynthetic responses on plant tolerance to adverse factors, revealing methods of their modification is an important scientific task. Earlier, we showed that a preliminary treatment of leaves of pea (*Pisum sativum* L.) by exogenous abscisic acid (ABA) decreases the amplitude of VP and the magnitude of response of the photosynthetic CO_2_ assimilation [33]. These effects seem to be caused by decreasing the initial H^+^-ATPase activity and initial A_hv_, which is dependent on the activity [10,33]. Based on these results, it can be expected that the preliminary treatment by other phytohormones, which are related to photosynthetic activity, can also modify ES-induced photosynthetic responses and, maybe, electrical signals. 

Brassinosteroids (particularly, 24-epibrassinolide, EBL) are plant steroid hormones participating in plant growth and development, elongation and division of cells, stress responses, photosynthetic regulation, and other processes [62,63,64,65]. Particularly, it is known that treatment by exogenous EBL influences photosynthetic processes in cucumber (*Cucumis sativus*) [66,67], maize (*Zea mays*) and spinach (*Spinacia oleracea*) [68], potato (*Solanum tuberosum*) [69], tomato (*Solánum lycopérsicum*) [70], wheat (*Triticum aestivum L.*) [71], brown mustard (*Brassica juncea*) [72], and pea [73]. It means that EBL can potentially influence photosynthetic responses induced by ESs in plants and, therefore, can be used as a tool for control of plant tolerance to adverse factors through modification of the influence of electrical signals on photosynthesis. 

The aim of the current work was to investigate the influence of preliminary treatment by exogenous EBL (spraying) on the burning-induced electrical signals and photosynthetic and transpiration responses in pea plants. The local burning by open flame (see Section 4.2 for details) was used as the typical inductor of VP [8,10]. 

## 2. Results

### 2.1. Influence of EBL Treatment on Photosynthetic Parameters Without Induction of Electrical Signals

At the first stage of the investigation, we revealed the EBL concentration, which was used in further investigation. It is known that EBL can increase the plant tolerance to heating [65,71] and that 0.01–2 µM concentrations of the 24-epibrassinolide are often used in photosynthetic investigations [66,67,68,70,71,72,73]. Considering these points, we investigated the influence of 0.01, 0.1, and 1 µM concentrations of EBL on tolerance of the photosynthetic CO_2_ assimilation to the action of increased temperature (whole-plant heating from 23 to 24 °C to 48 °C for 30 min) to reveal EBL concentrations modifying this tolerance. 

**Figure 1:**
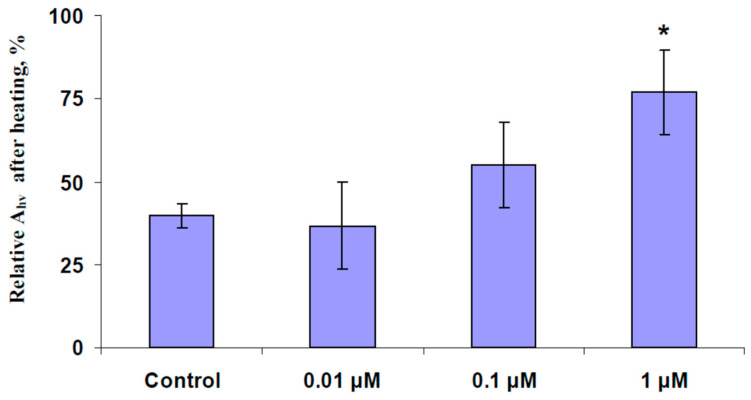
The influence of treatment by different concentrations of the exogenous 24-epibrassinolide (EBL) on relative values of the photosynthetic CO_2_ assimilation (A_hv_) after the heating of pea (*Pisum sativum* L.) (*n* = 5–15). Experimental plants were sprayed with the water solution with 24-epibrassinolide (0.01, 0.1, and 1 µM) 1 day before heating of the pea plants. The control plants were sprayed with water without EBL. A_hv_ was calculated as the A + R, where A was the measured CO_2_ assimilation and R was the dark respiration. A_hv_ was estimated before the pea plant heating and 1 day after. Relative values of A_hv_ were calculated as ratios between photosynthetic assimilations after and before heating. *, control and experimental values are significantly differed (*p* < 0.05).

It was shown (Figure 1) that the action of increased temperature strongly suppressed A_hv_ because the residual CO_2_ assimilation 1 day after heating was about 40% from its initial value. Preliminary spraying by 0.01 and 0.1 µM concentrations of EBL did not significantly influence the residual A_hv_. In contrast, the spraying of 1 µM concentration of EBL significantly increased the residual A_hv_ which was about 77% from the initial value of the photosynthetic CO_2_ assimilation. As a result, 1 µM EBL concentration, which positively influenced pea heat tolerance, was used in this investigation. 

**Figure 2:**
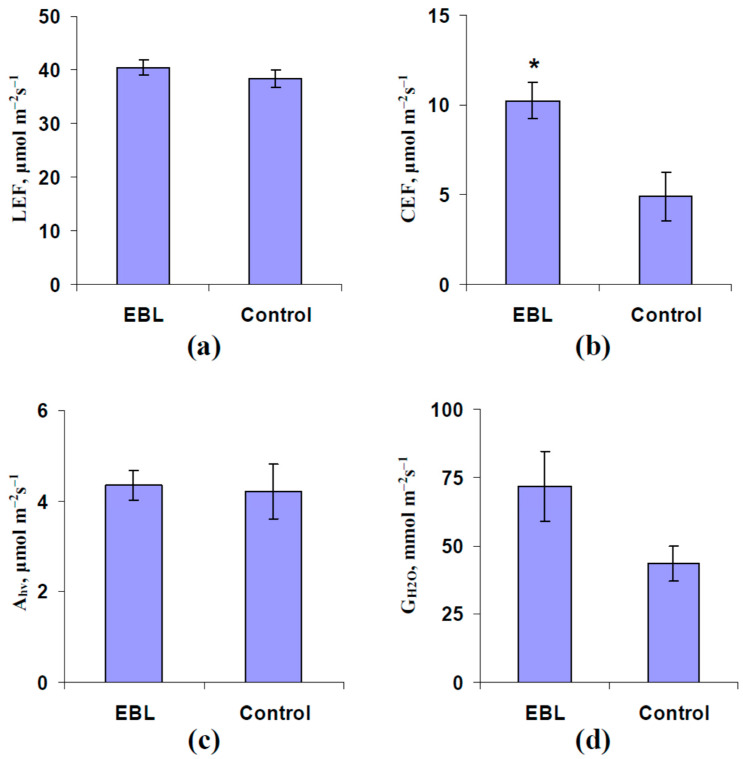
The influence of treatment with exogenous 24-epibrassinolide (EBL) on the photosynthetic linear electron flow (LEF) (**a**), cyclic electron flow around photosystem I (CEF) (**b**), photosynthetic CO_2_ assimilation (A_hv_) (**c**), and stomatal water conductance (G_H2O_) (**d**) without induction of electrical signals in pea plants (*n* = 5–7). The experimental plants were sprayed with the water solution with 1 µM 24-epibrassinolide 1 day before the photosynthetic measurements. The control plants were sprayed with water without EBL. The photosynthetic parameters were measured after the 150 min adaptation in the measuring system to correspond with the investigations of the electrical signals and photosynthetic responses in the current work. A_hv_ was calculated as the A + R, where A was the measured CO_2_ assimilation and R was the dark respiration. *, control and experimental values are significantly differed (*p* < 0.05).

Investigation of the influence of the EBL treatment (1 µM) on photosynthetic and transpiration parameters in pea plants without the induction of electrical signals by the local burning showed that LEF, A_hv_, and G_H2O_ were not significantly changed after spraying by the 24-epibrassinolide (Figure 2). The result was in good accordance with the variability of the EBL influence on photosynthetic processes in plants [65]. 

In contrast, the EBL treatment significantly stimulated CEF (Figure 2b); the value of CEF was about 200% compared to the control. Considering the participation of CEF in plant adaptation to the action of stressors [74,75], the increasing cyclic electron flow could participate in increasing the pea plant tolerance to heating, which is shown above (Figure 1).

### 2.2. Influence of EBL Treatment on Burning-Induced Electrical Signals in Pea Plants

**Figure 3:**
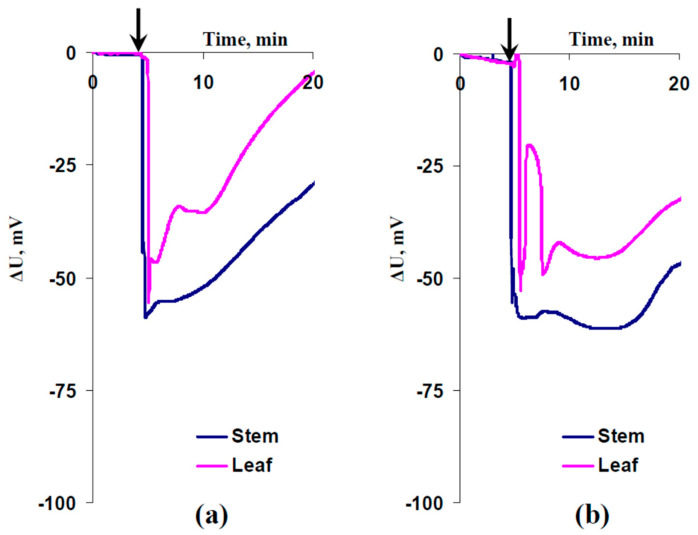
Records of changes in the surface potential (ΔU) in the second mature leaf and in the stem near this leaf were induced by the local burning of the first mature leaf (arrow). (**a**) Pea plants were treated with 1 μM exogenous 24-epibrassinolide (EBL) 1 day before the electrical measurements. (**b**) Plants were not treated with EBL. Tops of the first mature leaves were burned (2–3 s, flame) after the 150 min adaptation in the measuring system.

**Figure 4:**
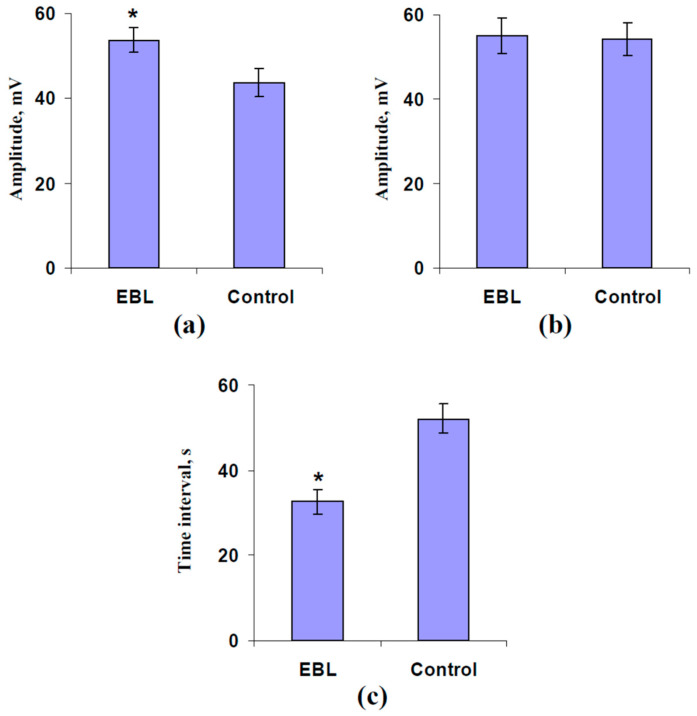
The influence of treatment with exogenous 24-epibrassinolide (EBL) on the amplitude of the burning-induced variation potential (VP) in the second mature leaf (**a**) and stem near the leaf (**b**) and on the time interval between initiations of these VPs (**c**) (*n* = 6–8). Experimental pea plants were sprayed with the water solution of 1 µM 24-epibrassinolide 1 day before electrical measurements. The control plants were sprayed with water without EBL. Tops of the first mature leaves were burned (2–3 s, flame) after the 150 min adaptation in the measuring system. *, control and experimental values are significantly differed (*p* < 0.05).

Figure 3 and Figure 4 show records of ESs and their average parameters, respectively. These signals were induced by the local burning of the first mature leaves 1 day after spraying with EBL (1 µM) or water without 24-epibrassinolide. It was shown (Figure 3) that burning caused electrical signals with an irregular shape and large duration, which could be classified as variation potentials [2,3,8,10,45]. 

The records also showed that the delay between the generation of electrical signals in the leaf and the stem was lower after the EBL treatment (see Figure 3a,b). Analysis of the average parameters of ESs supported this result. It was shown that the EBL treatment significantly increased the VP amplitude in the leaf (Figure 4a) and decreased the time interval between ES generation in the stem and the leaf (Figure 4c). 

Thus, our results showed that the EBL treatment contributed to the variation potential propagation from the stem to the leaf. Based on these results, it could be expected that the EBL treatment should also stimulate the photosynthetic and transpiration responses in pea plants. 

### 2.3. The Influence of EBL Treatment on Burning-Induced Photosynthetic and Transpiration Responses in Pea Plants

**Figure 5:**
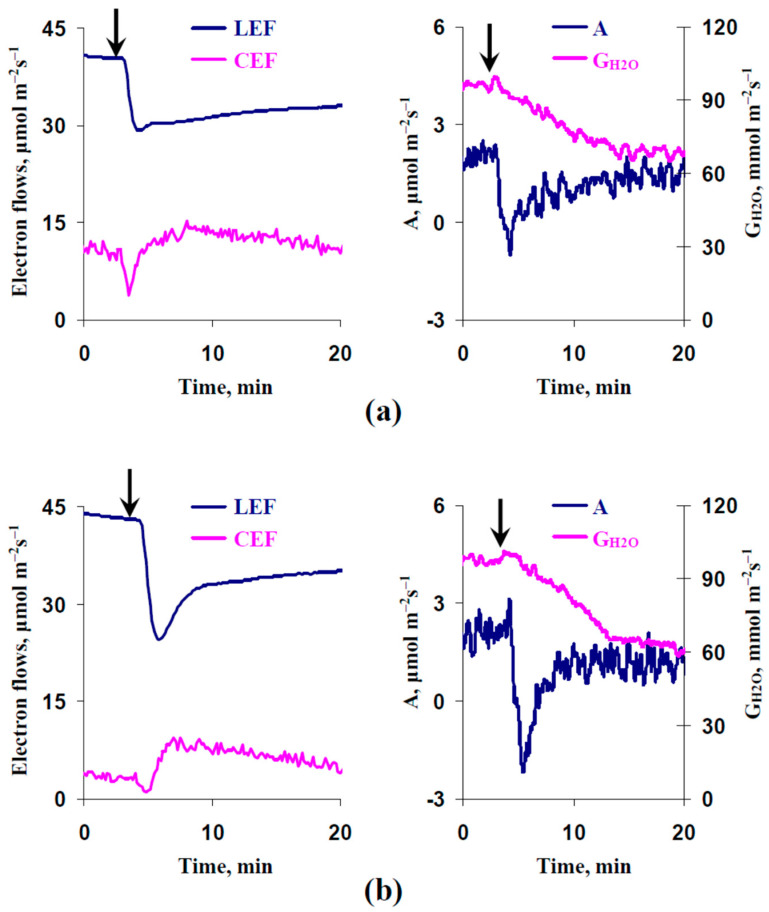
Records of changes in the photosynthetic linear electron flow (LEF), cyclic electron flow around photosystem I (CEF), measured CO_2_ assimilation (A), and stomatal water conductance (G_H2O_) in the second mature leaf induced by the local burning of the first mature leaf (arrow) and the ES propagation. (**a**) Pea plants were treated with 1 μM exogenous 24-epibrassinolide (EBL) 1 day before the photosynthetic measurements. (**b**) Plants were not treated with EBL. Tops of the first mature leaves were burnt (2–3 s, flame) after the 150 min adaptation in the measuring system.

Figure 5 shows that the local burning-induced transient photosynthetic and transpiration inactivation included a decrease in the photosynthetic linear electron flow (LEF), measured CO_2_ assimilation (A), and stomatal water conductance (G_H2O_), and an increase in the cyclic electron flow around photosystem I (CEF). These responses were in good accordance with numerous experimental investigations, which showed the fast photosynthetic inactivation induced by ESs (see, e.g., our reviews [8,10]). 

It should be additionally noted that ES-induced changes in CEF included two components: the primary fast decreasing and secondary slow increasing. This result was in accordance with our previous work [29]. The EBL treatment stimulated the ES-induced primary decreasing CEF (Figure 5a). 

**Figure 6:**
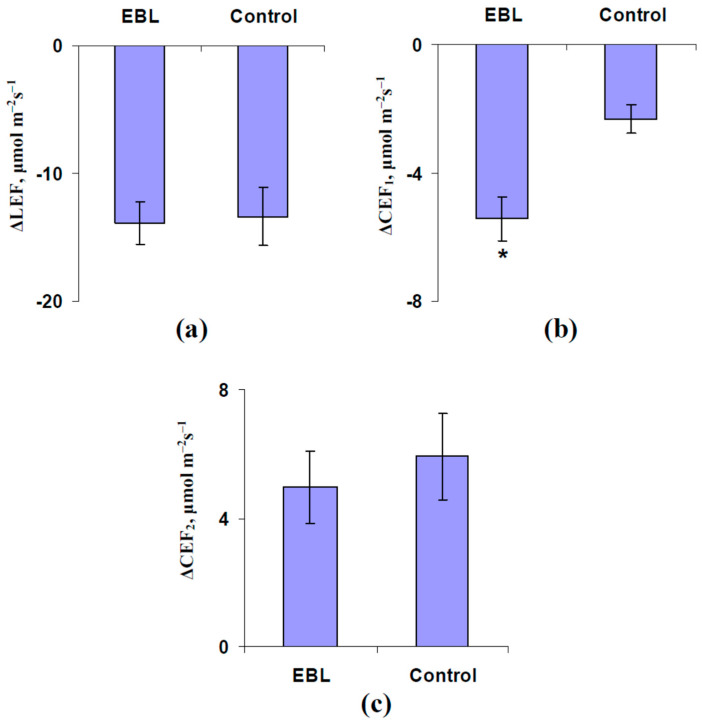
The influence of treatment with exogenous 24-epibrassinolide (EBL) on the magnitudes of decreasing LEF (ΔLEF) (**a**), primary decreasing CEF (ΔCEF_1_) (**b**), and secondary increasing CEF (ΔCEF_2_) (**c**) in the second mature leaf induced by the local burning of the first mature leaf in pea plants and ES propagation (*n* = 5–7). Experimental plants were sprayed with the water solution of the 1 µM 24-epibrassinolide 1 day before photosynthetic measurements. The control plants were sprayed with water without EBL. The tops of the first mature leaves were burnt (2–3 s, flame) after the 150 min adaptation in the measuring system. *, control and experimental values are significantly differed (*p* < 0.05).

**Figure 7:**
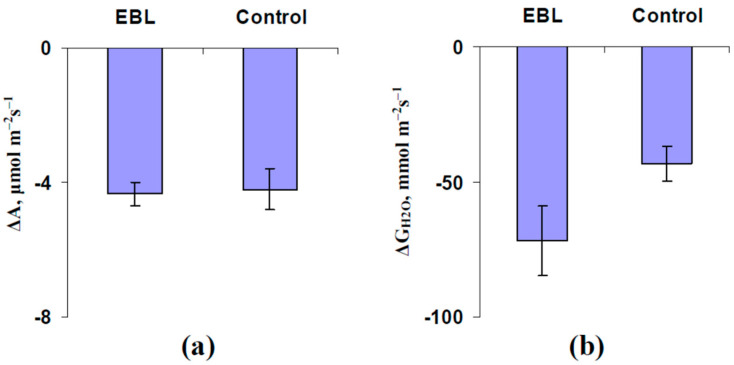
The influence of treatment with exogenous 24-epibrassinolide (EBL) on the magnitudes of decreasing A (ΔA) (**a**) and G_H2O_ (ΔG_H2O_) (**b**) in the second mature leaf induced by the local burning of the first mature leaf in pea plants and ES propagation (*n* = 6–7). Experimental plants were sprayed with the water solution of 1 µM 24-epibrassinolide 1 day before photosynthetic measurements. The control plants were sprayed with water without EBL. The tops of the first mature leaves were burnt (2–3 s, flame) after the 150 min adaptation in the measuring system. Significant differences were absent.

Analysis of the average values of the LEF and CEF (Figure 6) showed that the EBL treatment did not significantly influence the magnitudes of the LEF decreasing and secondary CEF increasing (Figure 6a,c). In contrast, the magnitude of the ES-induced primary decreasing LEF was significantly increased (Figure 6b). Analysis of A_hv_ (Figure 7a) and G_H2O_ (Figure 7b) was not shown to influence the EBL treatment on the magnitudes of ES-induced changes in these photosynthetic parameters. 

**Figure 8:**
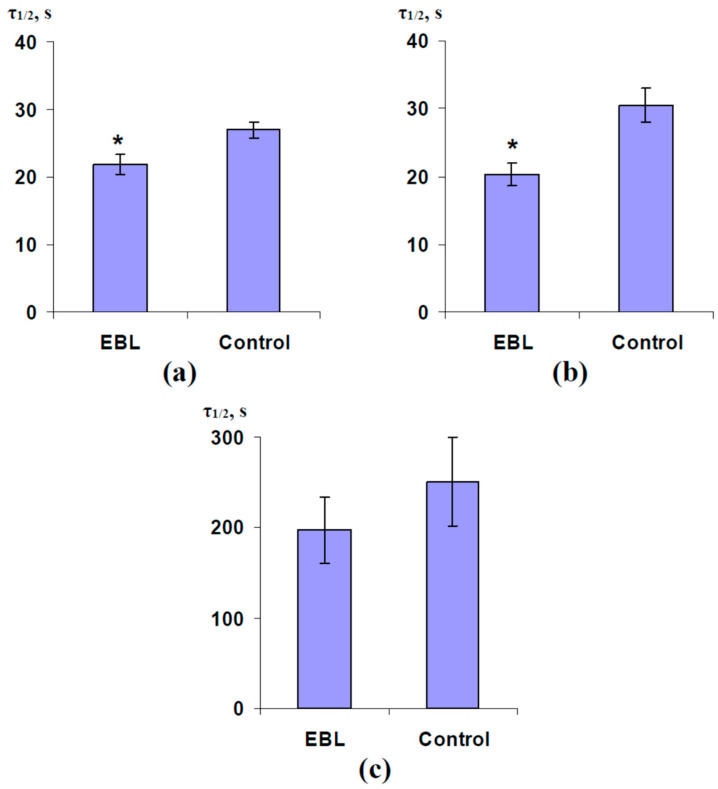
The influence of treatment with exogenous 24-epibrassinolide (EBL) on the duration of 50% decreasing (τ_1/2_) LEF (**a**), A (**b**), and G_H2O_ (**c**) in the second mature leaf induced by the local burning of the first mature leaf in pea plants and ES propagation (*n* = 5–7). Experimental plants were sprayed with the water solution of 1 µM 24-epibrassinolide 1 day before photosynthetic measurements. The control plants were sprayed with water without EBL. The tops of the first mature leaves were burnt (2–3 s, flame) after the 150 min adaptation in the measuring system. τ_1/2_ of CEF changes were not analyzed because these changes were intricate (decreasing and following increasing). *, control and experimental values are significantly differed (*p* < 0.05).

Figure 8 shows the average duration of 50% decreasing (τ_1/2_) LEF, A, and G_H2O_, which were observed after the induction of ESs. In the control, these durations were about 30 s for the linear electron flow and CO_2_ assimilation (Figure 8a,b) and about 250 s for the stomatal water conductance (Figure 8c). The preliminary EBL treatment decreased τ_1/2_ (for changes in LEF and A) from 27 to 30 s to 20 to 22 s; in contrast, significant changes in τ_1/2_ for G_H2O_ were absent. These results showed that the EBL treatment could accelerate the formation of the photosynthetic response. 

Finally, in the small experimental series, we analyzed the influence of the EBL treatment on the time interval between the local burning and the initiation of the photosynthetic or transpiration changes in the non-irritated second mature leaf. It was shown (Figure S1) that this interval was significantly decreased for LEF, CEF, and A after the EBL treatment; in contrast, the treatment did not influence the time interval for G_H2O_. 

Thus, our results showed that the EBL treatment accelerated the formation of ES-induced photosynthetic responses in the non-irritated second mature leaf in pea plants. In contrast, the magnitudes of ES-induced photosynthetic responses were weakly affected by the phytohormone treatment (excluding primary decreasing CEF). The EBL treatment weakly influenced the parameters of ES-induced changes in G_H2O_.

## 3. Discussion

ESs are important stress signals that change physiological processes [2,3,6,7,8,10,37] to increase plant tolerance to action of adverse factors [8,10]. ES-induced photosynthetic and transpiration responses, which are related to the transient inactivation of H^+^-ATPase accompanying the generation of different types of electrical signals [10], are probable to play an important role in this tolerance increase [6,8,10]. It means that the development of methods to regulate ESs and ES-induced physiological responses (particularly, changes in photosynthesis and transpiration) is a prospective scientific problem; its solution can potentially provide new tools for the management of productivity and survival of agricultural plants under the action of adverse factors. 

Potentially, the treatment of plants by chemical agents modifying their electrical characteristics is an effective method to regulate ESs. Particularly, there are works [76,77] showing that treatment with genistein or menthol influences the characteristics of the action potentials induced by light or cold. The plant treatment by exogenous stress phytohormones is also a possible method for the regulation of ESs and ES-induced physiological responses. Earlier, we showed that the preliminary plant spraying with exogenous ABA decreases the amplitude of the burning-induced ESs and the magnitude of the following changes in A_hv_ [33]. In the current work, we analyze the influence of the preliminary treatment of plants by the exogenous EBL on electrical signals (variation potentials) and ES-induced photosynthetic responses. 

The preliminary EBL treatment (1 µM, spraying) increases the tolerance of pea photosynthesis to heating (Figure 1). In contrast, under favorable conditions, only CEF are affected by this treatment (Figure 2b); LEF, A_hv_, and G_H2O_ are not changed (Figure 2a,c,d). The positive influence of the EBL treatment on the pea plant heat tolerance is in accordance with numerous works [64,65,69,70,71] which show the increasing tolerance of plants to adverse factors including heating [65,71] under this treatment. 

It is known that the activation of CEF is a typical adaptive response protecting photosynthetic processes under the action of adverse factors [73,74]. This activation can decrease damage to the photosynthetic machinery under the action of stressors (including increased temperatures) through the suppression of the production of reactive oxygen species, the prevention of overreduction of photosystem I, and stimulation of the energy-dependent non-photochemical quenching [29,74,75]. Additionally, CEF provides ATP to repair the photosynthetic machinery after heating [78]. Considering this point, the CEF stimulation after the EBL treatment can participate in increasing the photosynthetic tolerance to heating. 

It should be noted that the EBL-induced CEF activation was earlier shown in thylakoids of pea plants [73]. Authors explained this effect through unstacking of the thylakoid membranes after EBR treatment of plants (spraying); this unstacking is also hypothesized to be directly related to the tolerance of the photosynthetic machinery to increased temperatures [73]. These results show that the CEF activation can be caused by a specific mechanism, which is probably weakly related to changes in LEF. Moreover, the initially increased CEF can facilitate its further increase under the action of stressors (e.g., heating) and under the following overreduction of the acceptor side of photosystem I, which stimulates CEF [29]. 

The absence of changes in LEF and A_hv_ after the preliminary EBL treatment does not seem to be extraordinary results because the EBL influence on the photosynthetic processes in plants can be strongly varied (see, e.g., review [65]). Particularly, spraying with 24-epibrassinolide does not influence A_hv_ in maize leaves but significantly decreases the photosynthetic CO_2_ assimilation in young spinach leaves [68]; significant differences are absent in mature leaves. In contrast, other investigations show that the EBL treatment can stimulate A_hv_ in cucumber [66,67], tomato [70], wheat [71], and brown mustard [72]. It should be additionally noted that G_H2O_ can be decreased [68], increased [71,72], or invariable [68,70] after the EBL treatment. 

In the next stages of the current investigation, we show that the preliminary EBL treatment contributes to ESs (VP) propagation from the stem to the leaf because the amplitude of the electrical signals in leaves increased (Figure 4a) and the duration of this propagation decreased (Figure 4c). These results are in accordance with the acceleration of forming ES-induced photosynthetic responses (changes in the linear electron flow and CO_2_ assimilation) in leaves (Figure 8a,b) and with decreasing the time interval between the burning and initiation of these responses (Figure S1). Considering the strong relation between the ES generation in the leaf and the initiation of the photosynthetic changes [8,26,79], the acceleration of the ES propagation into the leaf seems to be the main reason for the acceleration of forming photosynthetic responses. 

Mechanisms of the revealed effects are currently not clear. However, earlier we showed that the amplitude of the variation potential and magnitudes of ES-induced photosynthetic changes are strongly related to the initial activity of H^+^-ATPase in the plasma membrane [33,80]. The decrease in the initial activity contributes to the decreased amplitude of ESs and the magnitudes of photosynthetic changes; in contrast, the increased initial activity contributes to the increased amplitude of ESs and the magnitudes of photosynthetic changes. Decreasing the H^+^-ATPase activity seems to be the mechanism of influence of the ABA treatment on ESs and the photosynthetic changes [33] because ABA decreases this activity [33,81,82,83]. 

Based on these results, we hypothesize that the activation of H^+^-ATPase in the plasma membrane under the EBL treatment can be a mechanism to contribute to the ES propagation into the leaf and to accelerate the photosynthetic changes. It is known that the brassinolide treatment stimulates the elongation growth [64,84,85]; activation of H^+^-ATPase in the plasma membrane is considered a potential mechanism of this effect [86,87]. Additionally, there are works [86,87] that show the activation of the H^+^-ATPase by brassinosteroids.

The positive influence of the preliminary H^+^-ATPase activation on the amplitude of the variation potential and its propagation into the leaf is supported by our previous results which show that increasing the initial H^+^-ATPase activity is linearly and positively related to the amplitude of VP [80]. It is known that Ca^2+^ influx is the probable mechanism of the induction of electrical signals [10]. Assuming that the magnitude of this influx is approximately constant for similar conditions (same plant species, same types of local damage, and same distances from the damaged zone), we can expect similar magnitudes of the relative changes in the H^+^-ATPase activity. It means that the magnitudes of the absolute changes should be increased under the increased initial activity of this transporter (e.g., after the EBL treatment) and vice versa. 

These increased magnitudes of absolute changes in the H^+^-ATPase activity should increase the magnitudes of changes in intra- and extracellular pH because the H^+^-ATPase is a key mechanism of supporting proton concentrations in the apoplast and cytoplasm of plant cells [88,89]. It is known [10] that increasing pH in the apoplast and decreasing pH in the cytoplasm and chloroplast stroma and lumen is the main mechanism of ES-induced photosynthetic responses. It means that photosynthetic responses induced by electrical signals should also be stimulated and, probably, accelerated under the increased initial activity of the H^+^-ATPase in the plasma membrane after the EBL treatment. Increasing the magnitude of ES-induced inactivation of photosynthetic CO_2_ assimilation in pea plants after preliminary activation of the H^+^-ATPase by its activator (the fungal toxin fusicoccin) additionally supports this point [80]. 

Thus, our hypothesis corresponds to the literature data and can potentially explain the mechanisms of the EBL influence on ES and photosynthetic responses. However, checking this hypothesis requires future investigations providing intracellular measurements of the active component of the membrane electrical potential because changes in this component show changes in the H^+^-ATPase activity [33,80,83].

As a whole, our results show that the EBL treatment contributes to the photosynthetic regulation by electrical signals in pea plants. This effect should probably stimulate a positive influence of the electrical signals on the plant tolerance to adverse factors because the ES-induced photosynthetic changes are an important mechanism of increasing this tolerance [6,8,10]. Previously, we showed [33] that the preliminary ABA treatment decreases the amplitude of ESs and the magnitude of the photosynthetic response. These results can be the basis of the development of methods of regulating plant tolerance to adverse factors (particularly, temperature [17], or drought [43]) through the suppression or stimulation of the influence of ESs on photosynthesis. However, it should also be noted that the influence of both ABA and EBL treatments on the electrical signals and photosynthetic responses was investigated in pea plants only. Considering the similarity of the mechanisms of ES and photosynthetic responses in different plants (see, e.g., works [19,26,27,29,30,31,32], it can be proposed that these treatments can induce similar modifications in many plant species, but the proposition requires checking. Thus, the analysis of the revealed effects in plants of different species, which are cultivated under different growth conditions, is an important task for future investigations. 

## 4. Materials and Methods

### 4.1. Plant Materials and Treatments

Two to three-week-old pea plants (*Pisum sativum* L.) were investigated. Plants were hydroponically cultivated using a half-strength Hoagland–Arnon medium in the climatic chamber (Binder KBW 240, Binder GmbH, Tuttlingen, Germany) under 23 °C and 16 h photoperiod. Pea plants were cultivated in small vessels (10 plants per vessel). 

In accordance with [33], the plants were sprayed with the EBL water solution (Sigma-Aldrich, St. Louis, MO, USA) or distilled water (control). The spraying continued up to the full wetting of the leaves (about 20 mL per vessel). The pea plants were sprayed 1 day before the initiation of other experimental procedures. 

### 4.2. Local Burning and Investigation of Electrical Signals in Pea Plants

**Figure 9:**
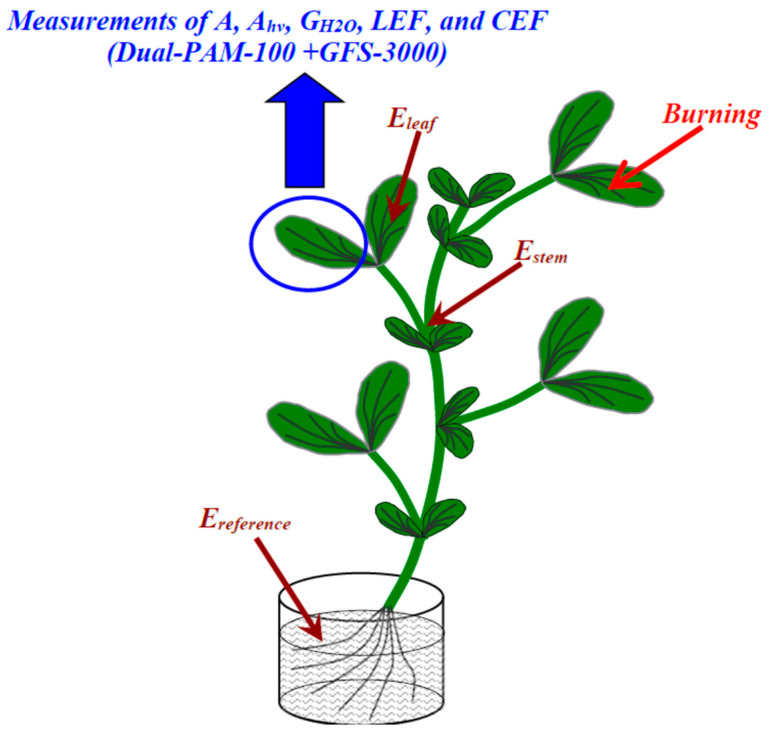
Scheme of measuring the electrical signals and photosynthetic responses after local burning (flame, 2–3 s) of the first mature leaf. E_leaf_ and E_stem_ are the measuring electrodes, which are placed on the second leaf (center of leaflet) and stem near this leaf, respectively. E_reference_ is the reference electrode. The photosynthetic parameters were measured by Dual-PAM-100 and GFS-3000. A, A_hv_, G_H2O_, LEF, and CEF are the CO_2_ assimilation, photosynthetic CO_2_ assimilation, stomatal water conductance, linear electron flow, and cyclic electron flow around photosystem I, respectively.

The local burning by open flame (duration was 2–3 s, size of the damaged zone was about 1 cm^2^) of the upper part of the first mature leaf was used to induce ESs (Figure 9) because the burning is a typical irritation causing VP [15,29,33]. The local burning occurred in 150 min after the fixation of pea plants to the measuring system. 

Extracellular measurements were used to investigate ESs. The system included Ag^+^/AgCl electrodes (Gomel Measuring Equipment Plant, Gomel, Belarus), a high-impedance IPL-113 amplifier (Semico, Novosibirsk, Russia), and a PC. The measuring electrodes were placed the second mature leaf and on the stem near this leaf (Figure 9), and the reference electrode was placed into a water solution surrounding the root. 

### 4.3. Measurements of Photosynthetic and Transpiration Parameters

In accordance with [33], the photosynthetic and transpiration parameters were measured in second mature leaves of the pea plants (Figure 9) using the standard system (Heinz Walz GmbH, Effeltrich, Germany), which included the GFS-3000 (gas analyzer), Dual-PAM-100 (PAM-fluorometer), and Dual-PAM gas-exchange Cuvette 3010-Dual (common measuring head). The CO_2_ concentration, humidity, and temperature in the measuring cuvette were 360 ppm, 20,000 ppm, and 23 °C, respectively. The 240 µmol m^−2^s^−1^ blue actinic light (460 nm) was used. 

Measurements of the parameters of the photosynthetic light reactions were initiated after the 15 min dark adaptation. Quantum yields of photosystem I (Φ_PSI_) and II (Φ_PSII_) were automatically estimated every 10 s under generation of saturation pulses using standard equations from [90,91], respectively. 

The linear electron flow (LEF) and cyclic electron flow around photosystem I (CEF) were calculated in accordance with our previous work [29]:

(1) $$\mathrm{LEF}=p\times \mathrm{PAR}\times \mathrm{dII}\times \Phi_{\mathrm{PSII}}$$

(2) $$\mathrm{CEF}=p\times \mathrm{PAR}\times \left(\mathrm{dII}\times \Phi_{\mathrm{PSII}}-\left(1-\mathrm{dII}\right)\times \Phi_{\mathrm{PSI}}\right)$$

where *p* = 0.88 (for pea leaves, [29,83]) is the fraction of the absorbed light in the total flux of the photosynthetically active radiation (PAR), and dII is the fraction distributed to photosystem II in the absorbed light. 

Equation (3) was used to calculate dII in accordance with our previous works [29,33]:

(3) $$\mathrm{dII}=\frac{{\Phi_{\mathrm{PSI}}}^{\mathrm{LL}}}{{\Phi_{\mathrm{PSI}}}^{\mathrm{LL}}+{\Phi_{\mathrm{PSII}}}^{\mathrm{LL}}}$$

where Φ_PSI_^LL^ and Φ_PSII_^LL^ were measured under the low light intensity of PAR (about 24 µmol m^−2^s^−1^).

GFS-3000 was used for measuring the CO_2_ assimilation (A) and stomatal water conductance (G_H2O_), which were automatically calculated by the GFS-3000 software (version 3.30). The photosynthetic CO_2_ assimilation (A_hv_) was calculated as A+R, where R was the dark respiration which was measured by GFS-3000 without illumination. 

### 4.4. Estimating Photosynthetic Tolerance to Heating

The photosynthetic heating tolerance was used as a criteria to select effective EBL concentration in the current work. The transient heating of the whole pea plants was used to estimate this tolerance (in accordance with [33]). The TV-20-PZ-“K” thermostat (Kasimov Instrument Plant, Kasimov, Russia) provided heating from 23 to 24 °C to 48 °C for 30 min. A_hv_ was measured before heating and 1 day after. Relative values of A_hv_ were calculated as ratios between photosynthetic assimilations after and before heating.

### 4.5. Statistics

Different pea plants were used for each experiment. Quantities of repetitions, average values, standard errors, and typical records are shown in the figures. The significance of differences was estimated using Student’s *t*-test. 

## 5. Conclusions

The generation and propagation of ESs are effective mechanisms to regulate photosynthetic activity and plant tolerance to stressors. It means that the development of methods of modification of ESs and ES-induced photosynthetic responses is an important scientific task because these methods can be potentially used for the management of productivity and survival of agricultural plants under adverse conditions. 

The current work was devoted to the analysis of the influence of the preliminary treatment (spraying) with exogenous 24-epibrassinolide on burning-induced ESs and following the photosynthetic and transpiration responses in pea plants. The investigation showed that the EBL treatment contributed to the ES propagation from the stem to the leaf and accelerated photosynthetic changes in the linear electron flow and CO_2_ assimilation induced by the local burning and the following ESs. Changes in the cyclic electron flow around photosystem I were also affected by the EBL treatment.

In the future, the revealed stimulation of the propagation of the electrical signals and acceleration of the photosynthetic changes by the EBL treatment can be the basis of the development of methods of increasing plant tolerance to the action of stressors. This development requires further investigations including the analysis of the mechanisms of influence of this treatment on ESs, on ES-induced photosynthetic responses, and, probably, on ES-induced changes in the plant tolerance to the action of adverse factors. 

## Data Availability

The original contributions presented in the study are included in the article’s Supplementary Materials, further inquiries can be directed to the corresponding author.

## Supplementary Materials

The following supporting information can be downloaded at: https://www.mdpi.com/article/10.3390/plants13233292/s1, Figure S1: Influence of treatment with exogenous 24-epibrassinolide (EBL) on the time interval between the local burning and initiation of changes in LEF, CEF, A, and G_H2O_ in the second mature leaf.
