# Supplementary material for: Preliminary Treatment by Exogenous 24-Epibrassinolide Influences Burning-Induced Electrical Signals and Following Photosynthetic Responses in Pea (Pisum sativum L.)
Source: Plants (Basel). 2024 Nov 23;13(23):3292. doi: 10.3390/plants13233292 (PMC11644061; doi:10.3390/plants13233292)
Supplement: Supplementary file 1 [file plants-13-03292-s001.zip › plants-3311193-supplementary.pdf]

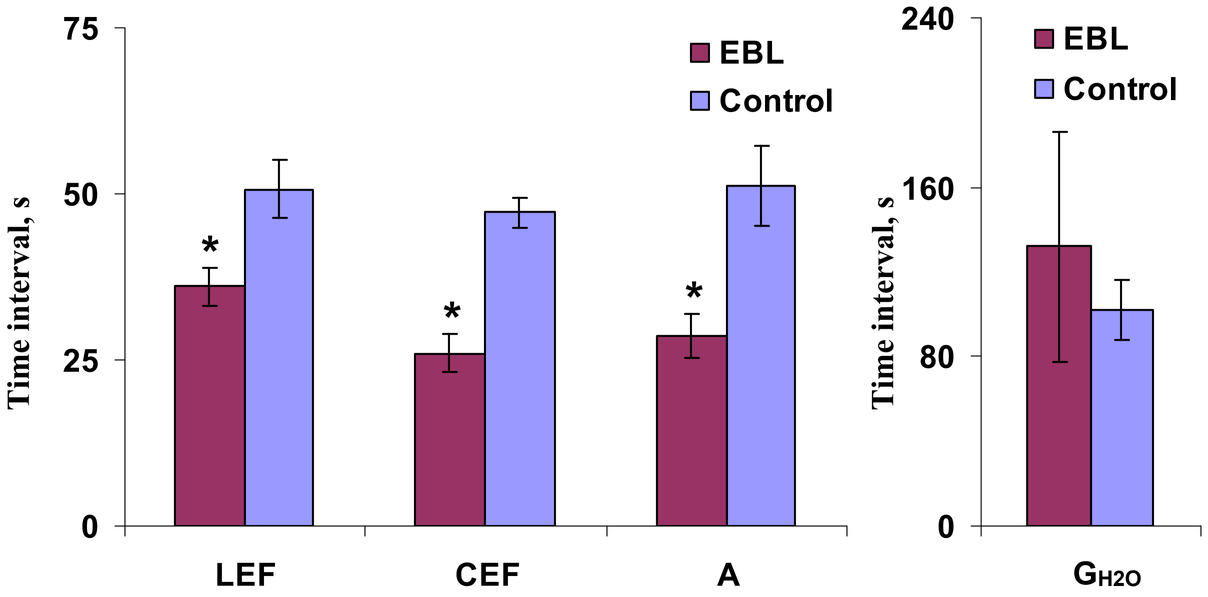

Figure S1. Influence of treatment by the exogenous 24-epibrassinolide (EBL) on the time interval between the local burning and initiation of changes in LEF, CEF, A, and G_{H2O} in the second mature leaf (*n*=4). Experimental plants were sprayed by the aqueous solution of the 1 μM 24-epibrassinolide in 1 day before photosynthetic measurements. Control plants were sprayed by aqueous solution. Tops of the first mature leaves were burned (2-3 s, flame) after the 150 min adaptation in the measuring system. *, control and experimental values are significantly differed (p<0.05).
